# Dietary Zinc Intake Affects the Association Between Dietary Vitamin A and Depression: A Cross-Sectional Study

**DOI:** 10.3389/fnut.2022.913132

**Published:** 2022-06-30

**Authors:** Biao Hu, Zheng-yang Lin, Run-pu Zou, Yin-wen Gan, Jia-ming Ji, Jing-xi Guo, Wan-gen Li, Yong-jing Guo, Hao-qin Xu, Dong-lin Sun, Min Yi

**Affiliations:** ^1^Department of Endocrinology, The Second Affiliated Hospital of Guangzhou Medical University, Guangzhou, China; ^2^Department of Clinical Medicine, The Second Clinical School of Guangzhou Medical University, Guangzhou, China; ^3^Department of Medical Imaging, The Second Clinical School of Guangzhou Medical University, Guangzhou, China; ^4^Department of Anesthesiology, The Second Clinical School of Guangzhou Medical University, Guangzhou, China; ^5^Guangzhou Medical University, Guangzhou, China

**Keywords:** cross-sectional study, vitamin A, depression, interaction, diet and nutrition

## Abstract

**Introduction:**

Dietary vitamin A concentrations correlate with depression. Zinc has been reported to be associated with lower depression. In addition, zinc is an important cofactor in the activation of vitamin A. However, there are few studies investigating relationships between of dietary zinc intake, dietary vitamin A intake and depression.

**Materials and Methods:**

The data for this study came from the National Health and Nutrition Examination Survey (NHANES) from 2005 to 2018 and involved 70,190 participants. We stratified participants by recommended dietary zinc intake (recommended dietary zinc intake for women: 8 mg/day, recommended dietary zinc intake for men: 11 mg/day). We further assessed the association between vitamin A and depression in participants with low and high zinc intake (interaction test) using univariate logistic regression of intake participants.

**Result:**

In the female population we grouped the population into low and high zinc intake groups using the recommended dietary zinc intake of 8 (mg/day), with an increase in total vitamin A, the risk of depression was significantly lower in the low zinc intake group (OR: 0.85 95 CI: 0.76–0.96), while the risk of depression was increased in the high zinc intake group (OR: 1.05 95 CI: 0.95 to 1.17). Thus, in the female population, there was a significant interaction between insufficient vitamin an intake and depression (interaction likelihood ratio test of *p* = 0.011). In the male population we grouped the population by the recommended dietary zinc intake of 11(mg/day). Again, the population was divided into two groups with low and high zinc intake, however we did not find significant results for the interaction (*p* = 0.743 for the interaction likelihood ratio test).

**Conclusion:**

Our findings suggest that zinc intake may influence the relationship between dietary vitamin A and depression. Of course, our findings require further randomized controlled trials to enhance the credibility.

## Introduction

In recent years, the increasing prevalence of depression has become a serious public health problem (1), which not only increases the associated morbidity and mortality, but in addition imposes a significant economic burden (2, 3). Therefore, it is essential to identify nutrients associated with depression to prevent its onset. Currently, vitamin A deficiency is becoming a common health problem (4). It is widely believed that vitamin A is an important raw material involved in the formation of light-sensitive substances within visual cells (5). In addition, vitamin A deficiency can cause dryness of the skin, conjunctiva and cornea, which can lead to severe dry eye disease and corneal ulcers, and this damage can involve epithelial tissues throughout the body, especially the respiratory, digestive and urinary tracts (6).

A possible link between vitamin A and depression has been identified in several recent research (7, 8). For example, a cohort study conducted by Bitarafan S showed a potential benefit of vitamin A intake in terms of reducing depression (7). In addition, a study conducted by Xue Y similarly showed that high intake of vitamin A significantly reduced the risk of depression (8). H In contrast, a study conducted by Hu P found that excessive vitamin A intake may increase the risk of depression and even suicidal tendencies (9). The discrepancy in the results of these studies may be due to inadequate consideration of potential confounding factors, like dietary zinc intake.

Zinc is an important regulator of the mammalian nervous and immune systems, a neurotransmitter of excitatory synapses in humans, and has an important role in stress responses and in the activation of zinc-dependent enzymes that maintain compensatory brain function (10). Previous studies have shown that zinc deficiency causes depression and anxiety-like behavior in humans, and that symptoms improve with zinc supplementation (11, 12). It has been suggested that zinc deficiency may also contribute to secondary vitamin A deficiency in the population, with a positive association between the two (13). However, clinical studies examining the effect of zinc intake on the association between vitamin A and depression are limited. In this cross-sectional study, we anticipated that zinc and vitamin A have an interaction effect on depression. The goal of this study was to investigate how zinc intake affected the link between vitamin A and depression.

## Materials and Methods

### Data Sources and Study Population

Data from the National Health and Nutrition Examination Survey (NHANES) conducted consecutively from 2005 to 2018 were used in this study. In our study, participants aged 47 years or older who underwent an interview and examination at a Mobile Examination Center (MEC) were included. Participants without relevant covariates, depression were excluded, and those without a complete 24-h dietary recall were not included. In the National Health and Nutrition Examination Survey, noninstitutionalized citizens of the United States are assessed in terms of their health and dietary habits. In order to select a representative sample of survey participants, a multistage stratified probability design was used (14). At the MEC, the program conducted in-depth interviews to collect demographic and health history information, performed physical examinations, and collected blood samples. The samples were analyzed at the National Center for Environmental Health, Laboratory Sciences Division of the Centers for Disease Control and Prevention.

The National Ethical Review Board for Health Statistics Research approved the study. The original study protocol (protocol #2005-06; #2011-17), duly approved by the Ethics Review Board, is available on the NHANES Ethics Review Board website (https://www.cdc.gov/nchs/nhanes/irba98.htm). Our research is based on publicly available data from NHANES, all details are from the official website (https://www.cdc.gov/nchs/nhanes/about_nhanes.htm).

### Measurement and Classification of Dietary Vitamin A and Zinc Dietary Intake

A dietary recall interview at the Mobile Examination Center (MEC)was used to collect information on zinc intake and vitamin A intake in the past 24 h. The dietary interview component is called What We Eat in America (WWEIA), and What We Eat in America data were collected using USDA's dietary data collection instrument, the Automated Multiple Pass Method (AMPM), available at: http://www.ars.usda.gov/nea/bhnrc/fsrg. The AMPM, providing accurate estimates of population intake (15, 16), was designed to provide an efficient and accurate means of collecting intakes for large-scale national surveys. The AMPM is a fully computerized recall method that uses a 5-step interview outlined below:

1. Quick List-Participant recalls all foods and beverages consumed the day before the interview (midnight to midnight).
2. Forgotten Foods - Participant is asked about consumption of foods commonly forgotten during the Quick List step.
3. Time and Occasion - Time and eating occasion are collected for each food.
4. Detail Cycle - For each food, a detailed description, amount eaten, and additions to the food are collected. Eating occasions and times between eating occasions are reviewed to elicit forgotten foods.
5. Final Probe - Additional foods not remembered earlier are collected.

All NHANES participants are eligible for two 24-h dietary recall interviews. The first dietary recall interview is collected in-person in the Mobile Examination Center (MEC) and the second interview is collected by telephone 3 to 10 days later. However, in order to ensure the accuracy of the data, the first dietary recall interview was chosen by our reach.

We stratified participants by recommended dietary zinc intake (recommended dietary zinc intake for women: 8 mg/day, recommended dietary zinc intake for men: 11 mg/day). Dietary vitamin A is a continuous variable, and its subgroups are grouped according to the median value. The decision to continue using this method in NHANES was based on consensus reached by a panel of experts at a regular workshop to evaluate NHANES data collection methods (17).

Zinc intake and vitamin A intake acquisition and measurement can be found in the NHANES database (https://wwwn.cdc.gov/Nchs/Nhanes/2005-2006/DR1IFF_D.htm).

### Depression Classification

Depression was defined based on PHQ-9 criteria and self-report questionnaires (18, 19).The Patient Health Questionnaire-9 (PHQ-9) is a self-rating scale for depressive disorders that is based on the nine entries of the DSM-IV (Diagnostic and Statistical Manual of Mental Disorders developed by the American Psychiatric Association) diagnostic criteria, and has high reliability and validity for both diagnosing depression and determining symptom severity. Depressed participants were defined as those who satisfied the following criteria: depression scores in the 0–4 range for not having depression and in the 5–15 range for having depression (18).

### Covariate

Age, gender, race/ethnicity, marriage, household income, body mass index (BMI), education level, smoking status, physical activity, work activity, alcohol consumption status, diabetes, and hypertension were considered as potential confounders in this study. Participants were self-classified on their race/ethnicity. Poverty Income Ratio (PIR) means a radio of family income to poverty, which can be found on the official website of NHANES (https://wwwn.cdc.gov/Nchs/Nhanes/2013-2014/DEMO_H.htm#INDFMPIR). Marital status was categorized as married or unmarried, where separated, divorced, cohabiting, married, and widowed were defined as married. Not having completed high school, having completed high school, and having completed college and above were the three levels of education. Current smokers, former smokers, and non-smokers were the three smoking categories. Current smokers were those who had smoked more than 100 cigarettes in the past and reported smoking for a few days or daily at the time of the interview. Ex-smokers were defined as people who had smoked more than 100 cigarettes in their lives but were no longer smokers. Nonsmokers were defined as those who had never smoked more than 100 cigarettes in their lives. According to the standardized protocol, BMI was calculated based on weight and height. Physical activity was classified into three levels according to the intensity of activity: non-work activity, moderate work activity and vigorous work activity. BMI was calculated based on measured height and weight. BMI was computed using height and weight measurements. On a digital scale, weight was measured in pounds and then converted to kilograms. Height was measured to the nearest millimeter using an electronic rangefinder.

If one of the following criteria is met, it can be judged as hypertension or diabetes. The definition criteria of diabetes are as follows: (1) doctor told you to have diabetes (2) Self-reported diabetes for a long time (3) glycohemoglobin HbA1c (%) >6.5 (4) the fasting glucose (mmol/L) ≥7.0 (5) random blood glucose (mmol/L) ≥11.1 (6) 2-h OGTT blood glucose (mmol/L) ≥11.1 (7) Use of diabetes medication or insulin (8) diabetes at birth is considered type 1 diabetes. Hypertension case definitions are based on the International Society of High Blood Pressure standards and a self-reported questionnaire. Participants were identified as hypertensive if they met the following criteria: (1) current use of hypertension medication (2) based on accurate diagnosis by the physician (3) based on blood pressure measured in real-time ≥140/90 mmHg (4) self-reported questionnaire data showing physician's prior diagnosis of hypertension and current use of medication to lower blood pressure. (5) The diagnostic criteria for hypertension by ambulatory blood pressure monitoring (ABPM) were: mean blood pressure ≥130/80 mmHg within 24 h, daytime ≥135/85 mmHg, at night ≥120/70 mmhg. Alcohol Data on alcohol drinking (yes = a minimum of 12 alcoholic beverages every year vs. no = fewer than 12 alcoholic beverages per year) was obtained by questionnaire interviews.

### Statistical Analysis

The statistical software R (http://www.R-project.org, The R Foundation) was used to conduct all of the analyses. For the stratified sampling data extracted from the nhanes database we used the statistical methods of multiple logistic regression analysis, stratified analysis, and sensitivity analysis (https://wwwn.cdc.gov/nchs/nhanes/AnalyticGuidelines.aspx). A multivariate linear regression approach was used to investigate the link between vitamin A and depression. Depression values were assessed at various levels of zinc intake. Interactions between subgroups were examined by likelihood ratio tests. Ninety-five percentage confidence intervals (Cls) were calculated. The statistical significance level was set at 0.05. Continuous variables were expressed as mean and standard deviation (SD) or median and interquartile range (IQR) in descriptive analysis, and categorical variables as weighted percentages (%). To examine continuous and categorical variables, the chi-square test (categorical variables), *t*-test (normal distribution), and Kruskal-Wallis (skewed distribution) tests were used.

## Result

### Baseline Characteristics of the Study Population

**Figure 1:**
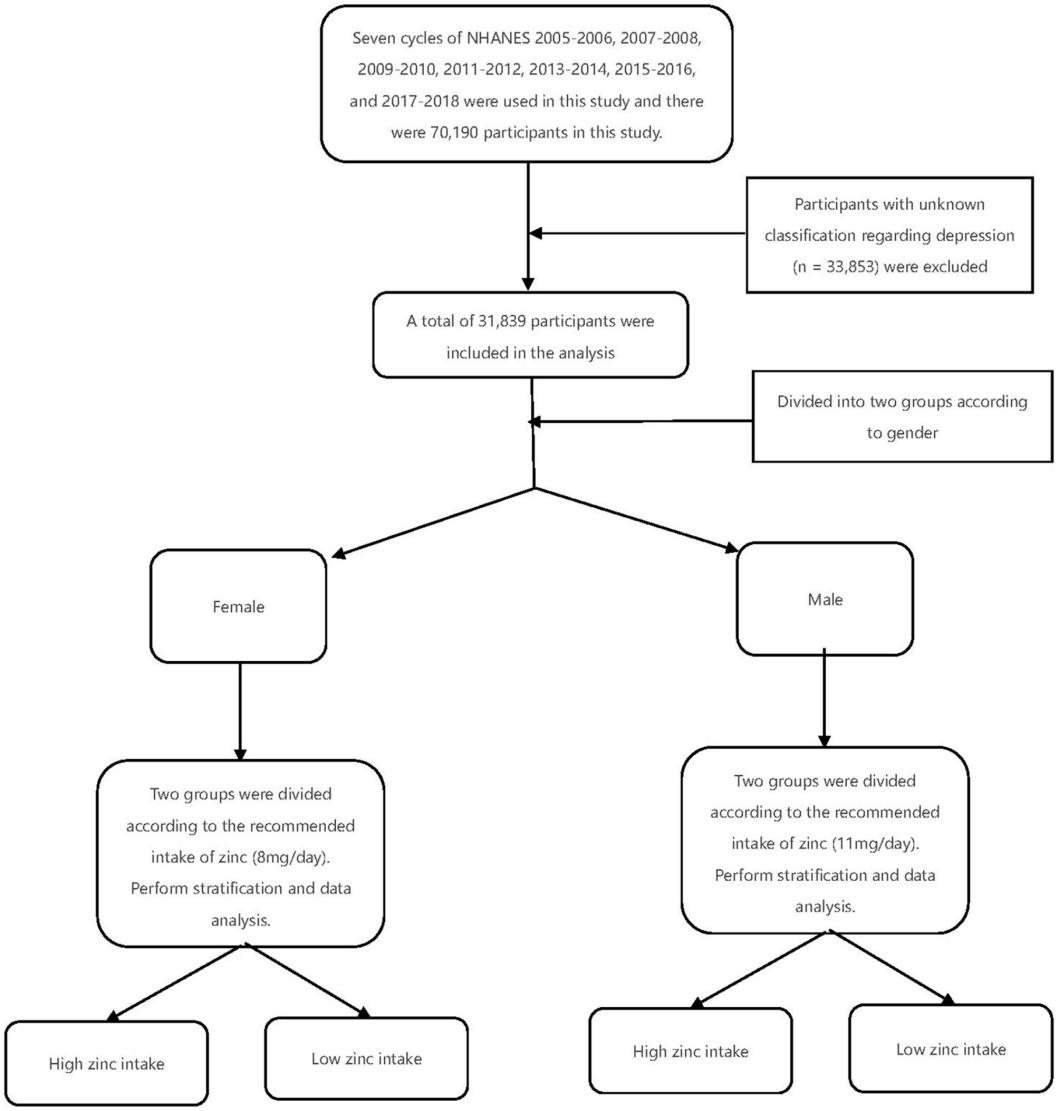
Experimental methods and procedures.

Seven cycles of NHANES 2005–2006, 2007–2008, 2009–2010, 2011–2012, 2013–2014, 2015–2016, and 2017–2018 were used in this study. There were 70,190 participants in this study, of whom 31,839 adults (≥18 years old) completed the interview, and our study also included the MEC examination. Participants with unknown classification regarding depression (*n* = 33,853) were excluded. After excluding participants with missing covariate data, a total of 31,839 participants were included in our analysis (Figure 1). A summary of the overall plot of exclusion criteria is shown in Figure 1. The descriptive characteristics of the participants according to their depression are shown in Table 1. Compared to the non-depression, participants with depression were more likely to be elder, male, non- Hispanic white, in the state of marriage, had higher BMI, had a higher level of education, PIR>1, lower intake of smoke, less physical activity, less work activity. There were no statistically significant differences in drinking status (*P* >0.05).

**Table 1:** Baseline characteristics of study participants.

	**Depressive state**			
**Variables**	**Total** **(*n* = 31,839)**	**Depression** **(*n* = 15,901)**	**Non-depression** **(*n* = 15,938)**	***p*-value**
Age, Median (IQR)	47.0 (31.0, 63.0)	47.0 (31.0, 63.0)	47.0 (31.0, 61.0)	0.004
Gender, *n* (%)				<0.001
Female	16,145 (50.7)	11,460 (47.7)	4,685 (60.1)	
Male	15,694 (49.3)	12,584 (52.3)	3,110 (39.9)	
Race, n (%)				<0.001
Mexican American	4,986 (15.7)	3,775 (15.7)	1,211 (15.5)	
Non-Hispanic black	6,851 (21.5)	5,166 (21.5)	1,685 (21.6)	
Non-Hispanic white	13,953 (43.8)	10,518 (43.7)	3,435 (44.1)	
Other Hispanic	2,845 (8.9)	2,038 (8.5)	807 (10.4)	
Other race-including multiracial	3,204 (10.1)	2,547 (10.6)	657 (8.4)	
Marital status, *n* (%)				<0.001
No	5,783 (18.2)	4,194 (17.4)	1,589 (20.4)	
Yes	24,693 (77.6)	18,841 (78.4)	5,852 (75.1)	
Unknown	1,363 (4.3)	1,009 (4.2)	354 (4.5)	
PIR, Mean ± SD	2.5 ± 1.6	2.7 ± 1.6	2.0 ± 1.5	<0.001
BMI, *n* (%)				<0.001
<25	9,494 (29.8)	7,412 (30.8)	2,082 (26.7)	
25–29.9	10,420 (32.7)	8,224 (34.2)	2,196 (28.2)	
>30	11,925 (37.5)	8,408 (20)	3,517 (45.1)	
Educational level, *n* (%)				<0.001
Less than high school	7,563 (23.8)	5,236 (21.8)	2,327 (29.9)	
High school graduation	7,601 (23.9)	5,615 (23.4)	1,986 (25.5)	
College or above	16,675 (52.4)	13,193 (54.9)	3,482 (44.7)	
Smoking status, *n* (%)				<0.001
Never	16,941 (53.2)	13,437 (55.9)	3,504 (45)	
Former	7,448 (23.4)	5,701 (23.7)	1,747 (22.4)	
Now	6,280 (19.7)	4,021 (16.7)	2,259 (21)	
Unknown	1,170 (3.7)	885 (3.7)	285 (3.7)	
Physical status, *n* (%)				<0.001
NO/Unknown	18,363 (57.7)	13,200 (54.9)	5,163 (66.2)	
Moderate	7,012 (22.0)	5,516 (22.9)	1,496 (19.2)	
Vigorous	6,464 (20.3)	5,328 (22.2)	1,136 (14.6)	
Work activity, *n* (%)				<0.001
Non-work activity	15,727 (49.4)	11,776 (49)	3,951 (50.7)	
Moderate work activity	6,076 (19.1)	4,545 (18.9)	1,531 (19.6)	
Vigorous work activity	5,563 (17.5)	4,159 (17.3)	1,404 (18)	
Unknown	4,473 (14.0)	3,564 (14.8)	909 (11.7)	
Alcohol, *n* (%)				0.119
No	7,635 (24.0)	5,814 (24.2)	1,821 (23.4)	
Yes	19,069 (59.9)	14,402 (59.9)	4,667 (59.9)	
Unknown	5,135 (16.1)	3,828 (15.9)	1,307 (16.8)	
DM, *n* (%)				<0.001
No	23,779 (74.7)	18,250 (75.9)	5,529 (70.9)	
Yes	8,060 (25.3)	5,794 (24.1)	2,266 (29.1)	
Hypertension, *n* (%)				<0.001
No	19,342 (60.7)	15,064 (62.7)	4,278 (54.9)	
Yes	12,497 (39.3)	8,980 (37.3)	3,517 (45.1)	
Vit A intake, Median (IQR)	465.0 (252.0, 770.0)	479.0 (262.0, 782.0)	427.0 (224.5, 731.5)	<0.001
Zn intake, Median (IQR)	9.7 (6.6, 14.1)	9.9 (6.8, 14.2)	9.2 (6.1, 13.7)	<0.001

### Association of Dietary Vitamin A With Depression

Among women, vitamin A was negatively associated with depression in the low-zinc group. However, in the high-zinc group, vitamin A was not associated with depression after adjusting for confounders (*p* > 0.05). This phenomenon was only observed in females.

### Zinc Intake Affects the Association Between Vitamin A and Depression

In the female population we grouped the population into low and high zinc intake groups using the recommended dietary zinc intake 8 (mg/day). The risk of depression was significantly reduced with increasing total vitamin A intake in the low zinc intake group (OR: 0.85 95 CI%: 0.76–0.96), while the risk of depression in the high zinc intake group (OR: 1.05 95 CI%:0.95–1.17), thus, there was a significant interaction between insufficient vitamin A intake and depression in the female population (The interaction likelihood ratio test was *p* = 0.011). In the male population, we grouped the population with the recommended dietary zinc intake of 11(mg/day), and similarly, divided the population into two groups with low and high zinc intake, however, we did not find a significant interaction (p=0.743 for the interaction likelihood ratio test). According to Table 2, the *p*-value of β remained stable, while in the high Zinc intake group, most of the *P*-values of β are not statistically significant in the low Zinc intake group.

**Table 2:** Effect of low and high zinc intake groups on the association between vitamin A and depression in dichotomous and trichotomous models.

Female (model 6)
**Variable**	**Dietary intake zinc≤8(mg/d)** **(*****n*** **=** **7,541)**		**Dietary intake zinc>8(mg/d)** **(*****n*** **=** **8,604)**		***P*** **for interaction**
**Vitamin A**	**OR(95 CI%)**	***P*** **-value**	**OR(95 CI%)**	***P*** **-value**	
**Subgroups**					0.011
≤ 440 (mcg)	1(reference)		1(reference)		
>440 (mcg)	0.85 (0.76~0.96)	0.007	1.05 (0.95~1.17)	0.331	
Male (model 6)
**Variable**	**Dietary intake zinc≤11(mg/d)** **(*****n*** **=** **7,397)**		**Dietary intake zinc>11(mg/d)** **(*****n*** **=** **8,297)**		**P for interaction**
**Vitamin A**	**OR(95 CI%)**	***P*** **-value**	**OR(95 CI%)**	***P*** **-value**	
**Subgroups**					0.743
≤ 495 (mcg)	1(reference)		1(reference)		
>495 (mcg)	0.97 (0.85~1.11)	0.679	1 (0.89~1.13)	0.952	

*Covariables include age, race, education level, PIR, marriage status, BMI, physical activity, work activity, smoking status, alcohol, DM and Hypertension*.

*Model 6: adjust for age, race, education level, PIR, marriage status, BMI, physical activity, work activity, smoking status, alcohol, DM, Hypertension*.

## Discussion

In our study, we found that vitamin A and low dietary zinc intake were significantly associated with a reduced prevalence of depression in a female population. This association remained significant after adjusting for confounding. In NHANES, the study sample size is quite large, and the quality is authoritative and strictly quality controlled. One-day of dietary intake data has been shown to be adequate for estimating and comparing mean intakes of population groups. (https://dietassessmentprimer.cancer.gov/profiles/recall) So our findings can be applied to all populations.

According to reports, depressed patients are commonly deprived of zinc (22, 23). And it has been reported that zinc intake is associated with a low prevalence of depression (23, 24). We divided the population by recommended dietary zinc intake into low and high zinc intake, and separated the results by gender. Our results suggest that zinc supplementation is effective in a range of depressed patients in the female population. However, it is worth noting that excessive zinc intake does not further reduce the risk of depression and does not hold for the male population, which is consistent with other reports (25, 26).

To our knowledge, only a few studies have looked at the effect of dietary zinc on the relationship between dietary vitamin A and depression. Similar to our findings, Qian Yao used the 24-item Hamilton Depression Scale to see if serum retinol-binding protein 4 (RBP4) concentrations may change depression symptoms in individuals (27). In the blood, RBP4 acts as a specific transporter protein for the micronutrient, vitamin A This research highlights the potential importance of adequate nutritional vitamin A status for adult brain function, owing to their role in the regulation of synaptic plasticity, as well as associated learning and memory behaviors, which may be a major factor in mood disorders such as major depression. The same conclusion was given by Farhadnejad H in his research (28). RBP4 can be transferred from the circulation system to the cerebro-spinal fluid and bind to retinol, thus facilitating the metabolism of retinol to retinoic acid in neuroepithelial cells (29). In turn, retinoic acid plays many important roles in regulating neurons, such as in plasticity, regeneration, differentiation, learning and memory (30).

According to our results, the relationship between zinc intake and depression is valid only in the female population and does not hold for men, which is in line with other reports (25, 26). Of course, the mechanisms involved have not yet been clearly elucidated. But in one study we learned of a possibility (26). First, according to current research, gender is an important factor affecting the clinical presentation and prognosis of depression (21, 31, 32). And according to the general research (33–35), the prevalence of depression is significantly higher in female than in male. This study considered that sex differences in neural structural and neurological functional parameters may be a factor associated with depressive symptoms of which gender differences in some serotonergic systems might play a role in the pathophysiology of depression (20). And it has been suggested that certain processes of the serotonin system may be more pronounced in women than in men (36, 37). This phenomenon may explain the interaction of zinc across genders in the relationship between vitamin A and depression.

However, a cross-sectional study showed that vitamin A can be an effective prevention (38), but not a treatment, for depression. It may be due to the study population with lower zinc intake. A study has shown that vitamin A and zinc deficiency usually occur at the same time because zinc deficiency reduces plasma retinol concentrations and reduces the production of retinol-binding proteins (39). Thus, zinc intake facilitates the potentiation of vitamin A activity. This impact could account for the interaction effect observed in our research. Given the nature of depression's etiological process, as well as the fact that depression is accompanied by an inflammatory response, including an increase in pro-inflammatory cytokines and lipid peroxidation (40), recent evidence supports an association between lipid peroxidation and major depression (41), and dietary antioxidants have the potential to play an important role in the prevention and treatment of depression. However, one study have shown that excessive intake of vitamin A can cause acute or chronic toxic effects (42). Of course, toxicity is only statistically possible after consuming 20 times more than the recommended intake of the vitamin and for several months. But perhaps it is the zinc intake that causes the increased activity of vitamin A, which lowers the threshold for vitamin A toxicity. Therefore, through our study, we believe that it is necessary to control the amount of zinc when using large amounts of vitamin A in the treatment of depression. This of course needs to be confirmed in further clinical trials.

In addition, one study found that venlafaxine treatment may reduce retinol binding protein 4 (RBP-4) levels. And the level of RBP4 in patients with major depressive depression (MDD) was lower than normal people (27). Therefore, it is necessary to take into account the effects of venlafaxine when considering the treatment of depression.

Our study still has some limitations. First, because of the cross-sectional design, we were unable to prove causality or directionality. Even after multiple adjustment, the results may be confounded by some other variables that were not measured. Nevertheless, some potential confounders were adjusted for in the logistic regression model, including some dietary factors. Second, there is no easy and exact way to measure total body zinc status. We obtained the zinc intake of participants by a dietary interview/24-h recall. Since dietary data were obtained from self-reported 24-h dietary recall, recall bias is difficult to avoid. Third, although a large sample was included in this study, the study population included only US residents. Therefore, practical considerations need to be taken into account when extrapolating to other populations. Therefore, well-designed multicenter controlled trials are needed to validate our findings.

## Conclusion

Conclusively, results of this study suggest that zinc intake may have an impact on the relationship between dietary vitamin A and depression. Despite providing clinical insight by this trial, more randomized controlled studies are required to provide more data.

## Data Availability Statement

NHANES has developed a public use dataset, available at: https://www.cdc.gov/nchs/nhanes/index.htm. Users can download relevant data for free for research and publish relevant articles. Our study is based on open source data, so there are no ethical issues and other conflicts of interest.

## Author Contributions

BH and Z-yL: conception and design and provision of study materials or patients. MY and D-lS: administrative support. Y-wG and R-pZ: collection and assembly of data. All authors: data analysis and interpretation, manuscript writing, and final approval of manuscript.

## Funding

The present study was supported by grant from National Natural Science Foundation of China (Grant No. 82000343) and grant from Natural Science Foundation of Guangdong Province (Grant No. 2019A1515110749).

## Conflict of Interest

The authors declare that the research was conducted in the absence of any commercial or financial relationships that could be construed as a potential conflict of interest.

## Publisher's Note

All claims expressed in this article are solely those of the authors and do not necessarily represent those of their affiliated organizations, or those of the publisher, the editors and the reviewers. Any product that may be evaluated in this article, or claim that may be made by its manufacturer, is not guaranteed or endorsed by the publisher.
